# Serum S100A8/A9 and MMP-9 levels are elevated in systemic lupus erythematosus patients with cognitive impairment

**DOI:** 10.3389/fimmu.2023.1326751

**Published:** 2024-01-25

**Authors:** Carolina Muñoz-Grajales, Michelle L. Barraclough, Juan P. Diaz-Martinez, Jiandong Su, Kathleen Bingham, Mahta Kakvan, Roberta Pozzi Kretzmann, Maria Carmela Tartaglia, Lesley Ruttan, May Y. Choi, Simone Appenzeller, Sherief Marzouk, Dennisse Bonilla, Patricia Katz, Dorcas Beaton, Robin Green, Dafna D. Gladman, Joan Wither, Zahi Touma

**Affiliations:** ^1^ Schroeder Arthritis Institute, Krembil Research Institute, University Health Network, Toronto, ON, Canada; ^2^ University of Toronto Lupus Clinic, Centre for Prognosis Studies in the Rheumatic Diseases, Toronto Western Hospital, Toronto, ON, Canada; ^3^ National Institute for Health and Care Research (NIHR), Manchester Biomedical Research Centre, Manchester University NHS Foundation Trust, Manchester Academic Health Science Centre, Manchester, United Kingdom; ^4^ Centre for Mental Health, University Health Network, Department of Psychiatry, University of Toronto, Toronto, ON, Canada; ^5^ Department of Medicine, Division of Neurology, University of Toronto Krembil Neurosciences Centre, Toronto, ON, Canada; ^6^ Department of Psychology, University Health Network-Toronto Rehabilitation Institute, Toronto, ON, Canada; ^7^ Cumming School of Medicine, University of Calgary, Calgary, AB, Canada; ^8^ School of Medical Science, Department of Orthopedics, Rheumatology and Traumatology, University of Campinas, São Paulo, Brazil; ^9^ Division of Rheumatology, Department of Medicine, and Institute for Health Policy Studies, University of California, San Francisco, Novato, CA, United States; ^10^ Institute for Work and Health, University of Toronto, Toronto, ON, Canada; ^11^ Department of Immunology, University of Toronto, Toronto, ON, Canada; ^12^ Department of Medicine, Division of Rheumatology, University of Toronto, Toronto, ON, Canada

**Keywords:** cognitive impairment, neuropsychiatric systemic lupus erythematosus (NPSLE), S100A8/A9, MMP-9 (matrix metalloproteinase 9), systemic lupus erythematosus

## Abstract

**Objective:**

Cognitive impairment (CI) is one of the most common manifestations of Neuropsychiatric Systemic Lupus Erythematosus (NPSLE). Despite its frequency, we have a limited understanding of the underlying immune mechanisms, resulting in a lack of pathways to target. This study aims to bridge this gap by investigating differences in serum analyte levels in SLE patients based on their cognitive performance, independently from the attribution to SLE, and exploring the potential for various serum analytes to differentiate between SLE patients with and without CI.

**Methods:**

Two hundred ninety individuals aged 18-65 years who met the 2019-EULAR/ACR classification criteria for SLE were included. Cognitive function was measured utilizing the adapted ACR-Neuropsychological Battery (ACR-NB). CI was defined as a z-score of ≤-1.5 in two or more domains. The serum levels of nine analytes were measured using ELISA. The data were randomly partitioned into a training (70%) and a test (30%) sets. Differences in the analyte levels between patients with and without CI were determined; and their ability to discriminate CI from non-CI was evaluated.

**Results:**

Of 290 patients, 40% (n=116) had CI. Serum levels of S100A8/A9 and MMP-9, were significantly higher in patients with CI (p=0.006 and p=0.036, respectively). For most domains of the ACR-NB, patients with CI had higher S100A8/A9 serum levels than those without. Similarly, S100A8/A9 had a negative relationship with multiple CI tests and the highest AUC (0.74, 95%CI: 0.66-0.88) to differentiate between patients with and without CI.

**Conclusion:**

In this large cohort of well-characterized SLE patients, serum S100A8/A9 and MMP-9 were elevated in patients with CI. S100A8/A9 had the greatest discriminatory ability in differentiating between patients with and without CI.

## Introduction

Systemic lupus erythematosus (SLE) is an autoimmune disease that can affect multiple organs and systems, including the peripheral (PNS) and central nervous system (CNS). In SLE, compromise of the nervous system (NS) manifests with different neurological and psychiatric symptoms known as neuropsychiatric lupus (NPSLE) that can be further grouped as focal or diffuse (1). Cognitive Impairment (CI), with an estimated prevalence of 38% (95% CI: 33-43%) (2), is one of the most common diffuse CNS NPSLE syndromes (2–4). Symptoms of CI include decline in memory, thinking speed, attention, and planning abilities, which can have a significant negative effect on patients’ daily functioning, social participation, and health-related quality of life (HRQoL) (5–9). Despite recognition of the importance of CI in SLE, the underlying mechanisms remain poorly understood (10), and therefore knowledge of biomarkers for CI and the molecular pathways to target for treatment are lacking.

Studies have reported that SLE patients with different NPSLE syndromes had alterations in the CSF or the serum levels of various analytes, including cytokines (IL-10, IL-6, IFN-γ, TNF-α, TWEAK, S100B, and S100A8/A9, amongst others) and proteases (NGAL and MMP-9) that can affect intrinsic brain components (e.g., blood-brain barrier [BBB], the neurovascular interface, and resident microglia) leading to neuroinflammation (11–17). However, studies focused on CI are scarce; therefore, the role of these analytes in CI remains unknown.

Interestingly, some of these analytes have also been implicated in the pathogenesis of neuroinflammatory diseases characterized by cognitive decline, such as multiple sclerosis (MS; IFN-γ, IL-6, IL-10, MMP-9, S100 proteins, and TWEAK) (18–20) and Alzheimer’s disease (AD; IL-6, IL-10, NGAL, S100 proteins, and TNF-α) (21–23), suggesting that there may be common underlying mechanisms that contribute to CI in these conditions and that these analytes could be useful biomarkers for CI in SLE.

In this study, we investigated whether the serum levels of nine analytes, IL-6, IL-10, IFN-γ, NGAL, MMP-9, S100A8/A9, S100B, TNF-α, and TWEAK, differed between patients with and without CI from a large, well-characterized SLE cohort. Considering that a person’s cognitive function is governed by different anatomic brain areas, to some extent represented by the ACR-NB domains, variation in serum analyte levels according to the patient’s performance (impaired or not impaired) in each domain was assessed. The ability of these serum analytes to discriminate SLE patients with CI from those without impairment was also investigated.

## Materials and methods

### Subjects and data collection

Study participants were part of a larger ongoing longitudinal cognitive study in the Toronto Lupus Clinic at the Toronto Western Hospital/University Health Network (TWH/UHN) in which patients aged between 18-65 years old that fulfill the 2019 EULAR/ACR SLE classification criteria (24), with adequate fluency in English enabling completion of the cognitive tasks, were consented and serially recruited. The study was approved by the Research Ethics Board of the University Health Network (REB# 15-9582). This paper presents results from the baseline visit of individuals recruited between January 2016 and February 2020.

The demographic and clinical data were recorded on a standardized data retrieval form. SLE disease activity and disease damage were determined with the SLEDAI-2K (25) and the Systemic Lupus International Collaborating Clinics/American College of Rheumatology Damage Index (SDI) (26), respectively.

### Patient and public involvement

Patients or the public were not involved in the design, or conduct, or reporting, or dissemination plans of our research.

### Cognitive assessment and classification of cognitive status

The study participants’ cognitive assessments were performed and interpreted by psychometrists and neuropsychologists at the SLE Clinic through the modified comprehensive one-hour ACR-NB (27), as previously described (28), that includes 19 cognitive tests representing six cognitive domains (D): D1. Manual motor speed, D2. Simple Attention and Processing Speed, D3. Executive Functioning, D4. Verbal fluency, D5. Visual-spatial Construction, and D6. Learning and Memory.

Since data from D1 were missing in a large proportion of participants (n=43, 14.8%) that were unable to perform the tests (dominant and non-dominant hand tapping) due to hand pain or established joint deformities, the motor speed domain scores were excluded from the analysis. The ACR-NB scores across the remaining 5 domains were then standardized by age and sex to classify patients into 3 groups: cognitive impairment (CI; z-score of ≤-1.5 in two or more domains), indeterminate CI (z-score of ≤-1.5 in only one domain), and non-CI (z-scores in all domains > -1.5) (29). As previously described, a domain was defined as impaired if a z-score of ≤ -1.5 was obtained in at least one test in D2, D3, and D4, and two or more tests in D5 and D6 (29).

### Analyte measurement

At the time of the neuropsychological assessment, patients provided blood samples that were processed to obtain serum and stored at -80 C° until use. In preliminary experiments, the impact of sample storage time on analyte levels was assessed using either linear regression or Spearman’s rank correlation (in instances where the prerequisites for linear regression were not fulfilled) and shown not to significantly affect the concentrations of the studied analytes. Serum levels of MMP-9, S100B, S100A8/A9, and TWEAK were measured by ELISA using DuoSets (R&D Systems, Minneapolis, MN, USA). High sensitivity (hs) ELISA kits were utilized to measure serum levels of NGAL (R&D Systems) IL-6, IL-10, TNF-α (R&D Systems), and IFN-γ (ThermoFisher Scientific, Waltham, MA, USA). All measurements were performed in duplicate following the manufacturer’s instructions (See **Supplementary Table 1** for dilution, dynamic range, and assay sensitivity). The intra-assay coefficient of variation (CV) ranged from 2.75 to 3.95%. For analytes measured on different days (IL-6, MMP-9, and TNF-α), the inter-assay CV ranged from 10.35% to 14.56%.

### Other laboratory measurements

Erythrocyte sedimentation rate (ESR), hsC-reactive protein (hsCRP), anti-dsDNA, antiphospholipid antibodies (APLA: anti-cardiolipin, anti-B2 glycoprotein I, and lupus anticoagulant) and complement levels (C3 and C4) were measured as part of the patients’ routine clinical assessments through the UHN laboratory. C3-C4 and anti-dsDNA antibodies were from the day of the cognitive assessment while APLA were the closest result within 30 days of the cognitive assessment. For correlation analyses, the ESR and hsCRP results were standardized a priori.

### Statistical analysis

#### Comparisons amongst groups and correlations

For continuous data, the differences among groups were determined by a Mann-Whitney *U* test for two groups (impaired and non-impaired domain) or a Kruskall-Wallis test with *post hoc* Dunn’s test with Bonferroni correction for multiple comparison (CI, indeterminate CI, and non-CI). Notably, for the S100B data, which exhibited a high incidence of non-arbitrary zero values across all groups, we adopted a modified Kruskal-Wallis approach as outlined in a prior study (30). A Chi-Square test was used for categorical variables. The correlation between measurements was determined using Spearman’s rank correlation coefficient.

#### Regression analysis

The relationship between serum levels of the different analytes and study participants’ performance in each test domain was investigated by regression analyses, adjusting for sex, age, race, educational level, SLEDAI-2K, SDI, and use of antimalarials, glucocorticoids, biologics, or immunosuppressants. To better satisfy the assumptions of the regression model, particularly concerning the normality of residuals and homogeneity of variances, a logarithmic transformation was applied to all analytes.

#### Analysis of predictive ability

To evaluate and contrast the measured analytes’ ability to discriminate SLE patients with CI from patients without CI, a predictive model using logistic regression was performed. For this analysis, the data were randomly partitioned into a training set (70%) and a test set (30%). We then calculated the area under the receiver operating characteristic curve (AUC) for each analyte using the R package pROC (31). Values of the AUC in the training set were interpreted as having outstanding, excellent, good, fair, and poor performance, corresponding to values of 1.0–0.91, 0.90–0.81, 0.80–0.71, 0.70–0.60, or <0.60, respectively (32). To account for overfitting, we performed bootstrapping with 1000 iterations to estimate the AUC and confidence intervals in the test set. Additionally, for those analytes with good or above performance, optimal cut-off values to discern between CI and non-CI were obtained by Youden’s index using the R package OptimalCutpoints (33), and their sensitivity (Sn), specificity (Sp), positive and negative predictive values (PPV and NPV, respectively), and positive and negative likelihood ratios (LR+ and LR-, respectively) were calculated and contrasted.

All statistical analyses were performed using GraphPad Prism version 9.1.5 (GraphPad Software, San Diego, CA, USA) or RStudio version 1.3.1073 (Integrated Development Environment for R. RStudio, PBC, Boston, MA, USA).

## Results

Of the 290 SLE patients included in this study, n=116 (40%) had CI, 81 (28%) had indeterminate CI, and 93 (32%) had non-CI. As shown in **Supplementary Figure 1**, patients most frequently had impairment in D5 (learning and memory, n=138, 47%), followed by D3 (visual spatial construction, n=100, 34%), and then D2 (simple attention and processing speed, n=65, 22%). D4 (verbal fluency) and D6 (executive functioning) were impaired in less than 20% of the patients (16% n=47, and 10% n=30, respectively). Of the 116 patients with CI, 69 (59.4%) had two domains impaired, 31 had three domains (26.7%), and the same proportion (6.8%, n=8), had four or five domains impaired.

The demographic and clinical characteristics of the study population as a whole and for the three groups (CI, indeterminate CI, and non-CI) are displayed in **Table 1**. Most subjects were of female sex and White self-reported race, however; there were different proportions of individuals of White and non-White race (Chi-square p=0,006) in patients with and without CI, consequently, race was incorporated as a covariate in downstream analyses. At assessment, the median age, disease duration, clinical characteristics, SLEDAI-2K, and SDI were comparable among groups. Compared with the other groups a higher absolute proportion of SLE patients with CI had musculoskeletal, renal, and muco-cutaneous involvement; however, this did not achieve statistical significance (Chi-square p=0.138, p=0.29, and p=0.134, respectively). The frequency of hypocomplementemia, anti-dsDNA, and APLA did not differ among the groups, and the medications that patients were taking were mostly similar, the only exception being significantly higher use of azathioprine (AZA) in non-CI patients (Chi-square p=0.001), which is consistent with our previous study (34).

**Table 1:** Study population characteristics.

Variable	Study population (n= 290)	Non-CI (n= 93, 32%)	Indeterminate CI (n= 81, 27.9%)	CI (n= 116, 40%)
Sex: female *n* (%)	258 (89%)	77 (83%)	75 (92%)	100 (86%)
Age at assessment: median (IQR)	40.7 (30.9 - 51.7)	38.8 (31.3- 49.4)	40.8 (30.5-53.5)	42 (30.8 - 52)
Self-reported race: *n* (%)				
White	154 (53%)	61 (65.6%)	43 (53%)	50 (43%)
Black	58 (20%)	9 (9.6%)	14 (17%)	35 (30%)
Chinese	33 (11%)	10 (10.7%)	11 (13.6%)	12 (10%)
Other*	45 (15.5%)	13 (13.9%)	13 (16%)	19 (16%)
Disease duration in years: median (IQR)	12 (5-21)	12 (6-22)	13 (5-21)	11 (3-20)
SDI score: median (IQR)	0 (0-2)	0 (0-1)	0 (0-2)	1 (0-2)
Clinical manifestation and serology: *n* (%)				
Neuropsychiatric (No CI)	2 (0.6%)	0	0	2 (1.7%)
Vasculitis	3 (1%)	0	0	3 (2.5%)
Musculoskeletal	25 (8.6%)	4 (4.3%)	7 (8.6%)	14 (12%)
Renal**	35 (12%)	8 (8.6%)	9 (11%)	18 (15.5%)
Muco-cutaneous	30 (10.3%)	5 (5.3%)	9 (11%)	16 (13.8%)
Serositis	2 (0.6%)	1 (1%)	1 (1.2%)	0
Constitutional	1 (0.3%)	0	1 (1.2%)	0
Hematologic	20 (6.9%)	6 (6.4%)	9 (11%)	5 (4.3%)
Clinical SLEDAI-2K score: median (IQR)	2 (0-4)	2 (0-4)	2 (0-4)	2 (0-6)
Positive anti-dsDNA	109 (37.5%)	44 (47%)	28 (34.5%)	44 (38%)
Low C3	119 (41%)	47 (50%)	28 (34.5%)	44 (38%)
Low C4	36 (12.4%)	17 (18%)	9 (11%)	10 (8.6%)
APLA	44 (15%)	18 (19%)	8 (9.8%)	18 (16.4%)
Current medication use: *n* (%)				
Antimalarial	227 (78%)	73 (78%)	59 (72.8%)	94 (81%)
Glucocorticoids	139 (47%)	49 (52.6%)	34 (41.9%)	56 (48%)
Glucocorticoid dose (prednisone or equivalent): median (IQR)	5 mg (4-10)	5 mg (4-10)	5 mg (4-11.25)	6 mg (2.6-10)
ACE inhibitor or Angiotensin receptor blockers	61 (21%)	19 (20%)	14 (17%)	28 (24%)
Immunosuppressant: *n* (%)	164 (56.5%)	53 (56.9%)	44 (54%)	69 (59%)
Azathioprine Methotrexate Mycophenolate Other	49 (29.8%) 26 (15.8%) 84 (51.2%) 5 (3%)	26 (49%) 6 (11.3%) 19 (35.8%) 2 (3.7%)	8 (18%) 9 (20.4%) 27 (61%) 0 (0%)	15 (21%) 11 (16%) 38 (55%) 5 (7.2%)
Biologics	12 (4%)	6 (6.4%)	4 (4.9%)	5 (4.3%)

SLEDAI-2K, Systemic Lupus Erythematosus Disease Activity Index-2000; SDI, Systemic Lupus International Collaborating Clinics/American College of Rheumatology Damage Index; APLA, antiphospholipid antibodies; ACE, angiotensin-converting enzyme. *Other: Native North American, Filipino, Mixed. ** Renal: as per SLEDAI definition (urinary cast, hematuria, proteinuria, or pyuria). The clinical manifestations, C3-C4 and anti-dsDNA antibodies were from the day of the cognitive assessment while APLA were the closest result within 30 days of the cognitive assessment.

### Comparison of serum analyte levels in SLE patients with and without CI

**Figure 1:**
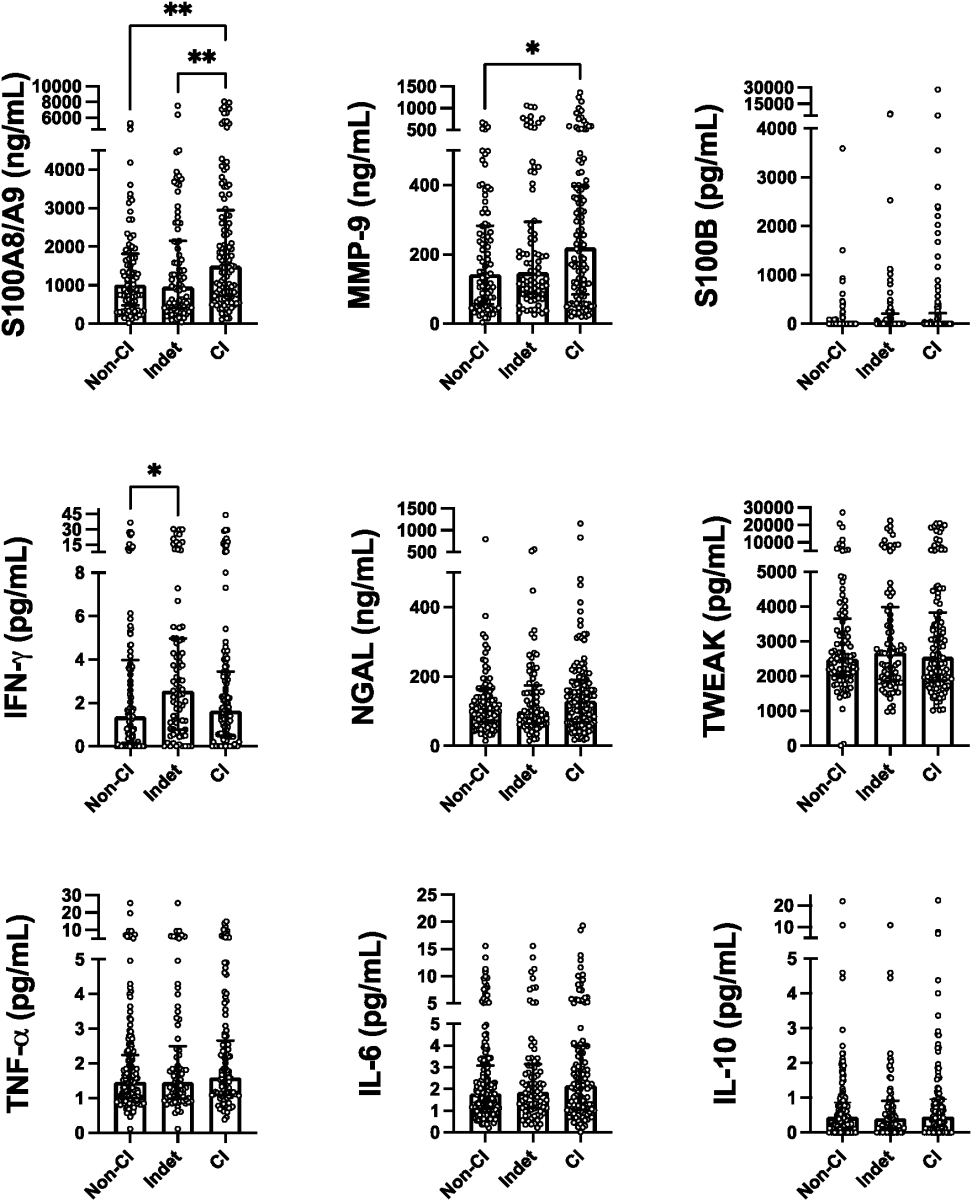
Serum levels of the measured analytes discriminated by patients' cognition status. Strip plots with median bars showing, from left to right, results for patients with non-Cl, Indeterminate CI (Indet), and definitive Cl. Each circle represents a single subject, with the top of the bar indicating the median for the subjects and error bars denoting the interquartile ranges. Statistical significance was determined using the Kruskal-Wallis test with Dunn's post-test for multiple comparisons with significant differences indicated by asterisks (*p ≤ 0.05, **p < 0.01).

Comparison of serum analyte levels among patients with CI (z-score ≤-1.5 in two or more domains), indeterminate CI (z-score ≤-1.5 in only one domain), and non-CI (z-scores in all domains >-1.5), as determined by Kruskall-Wallis, showed that compared to no-CI patients those with CI had significantly higher levels of S100A8/A9 (adjusted p=0.006, small effect size [eta^2 =^ 0.0383]) and to a lesser extend MMP-9 (adjusted p=0.036, small effect size [eta^2 =^ 0.014]). Similarly, compared to patients with indeterminate CI they had significantly higher S100A8/A9 levels (adjusted p=0.008). In the comparison between the indeterminate CI group and the non-CI group, levels of IFN-γ were significantly higher in the former (adjusted p=0.023, small effect size [eta2 = 0.019]). There were no significant differences in the levels of the other analytes amongst groups. These findings are presented in **Figure 1**.

### Within ACR-NB domain comparisons of serum analyte levels

**Figure 2:**
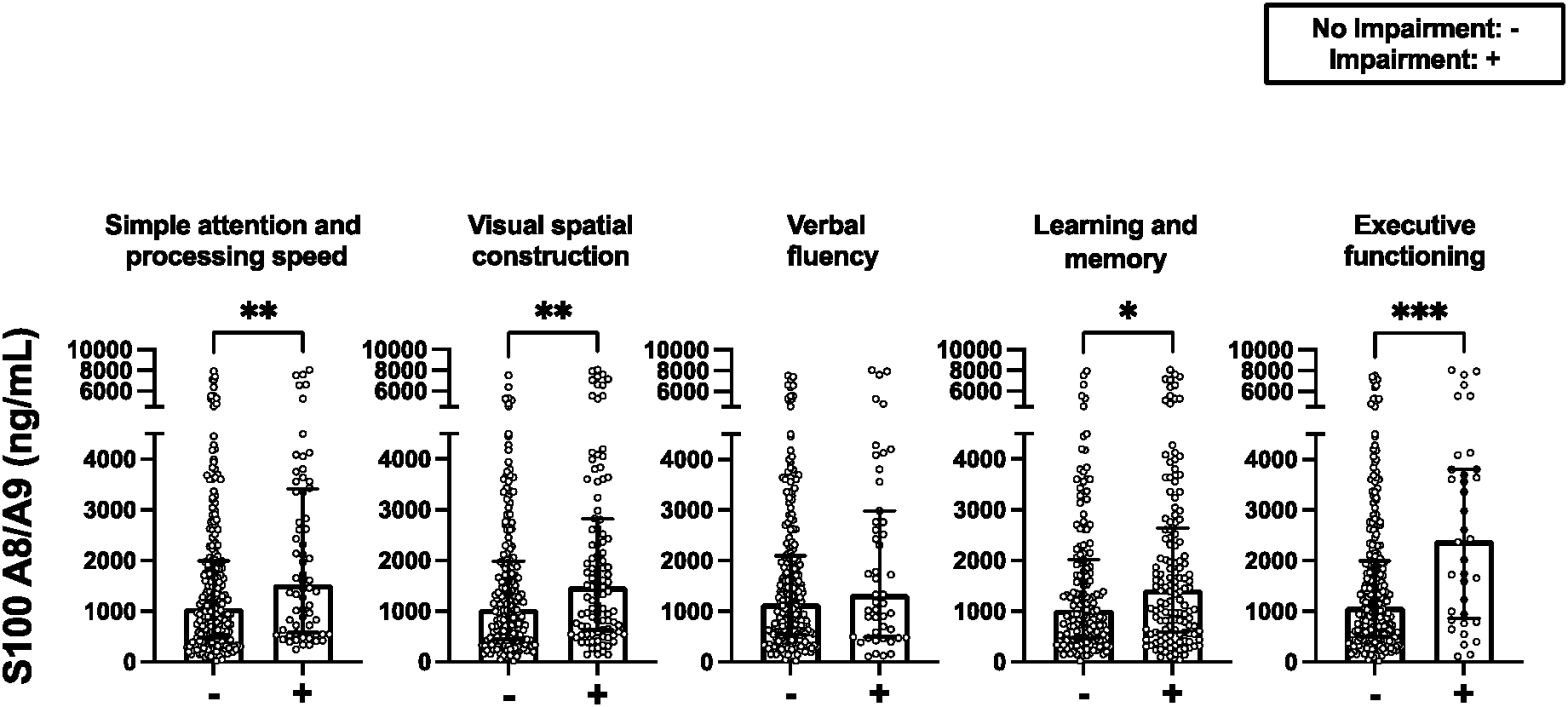
Serum levels of S100A8/A9 discriminated by the ACR-NB' domains. Strip plots with median bars showing, from left to right, results for patients with no impairment and impairment (z-score of ≤ -1.5 in at least one test in D2, D3, and D4, and two or more tests in D5 and D6) in the domain. Each circle represents a single subject, with the top of the bar indicating the median for the subjects and error bars denoting the interquartile ranges. Statistical significance was determined using the Mann-Whitney *U* test with significant differences indicated by asterisks (*p ≤ 0.05, **p ≤ 0.01 , ***p ≤ 0.001).

Across most domains, patients with impairment (z-score of ≤ -1.5 in at least one test in D2, D3, and D4, and two or more tests in D5 and D6) had significantly higher levels of S100A8/A9 compared to those without impairment (**Figure 2**). Additionally, in the domain of simple attention and processing speed (D2), patients with impairment displayed significantly elevated levels of IL-6 and TNF-α (p=0.010 and p=0.017, respectively) (refer to **Supplementary Figure 2**). However, when examining the remaining analytes, no significant differences in levels were observed between patients with and without impairment when stratified by domain (refer to **Supplementary Figure 3**). Similarly, although modest, only the levels of S100A8/A9 showed a positive correlation with the number of impaired domains (ρ=0.23, p< 0.001).

**Figure 3:**
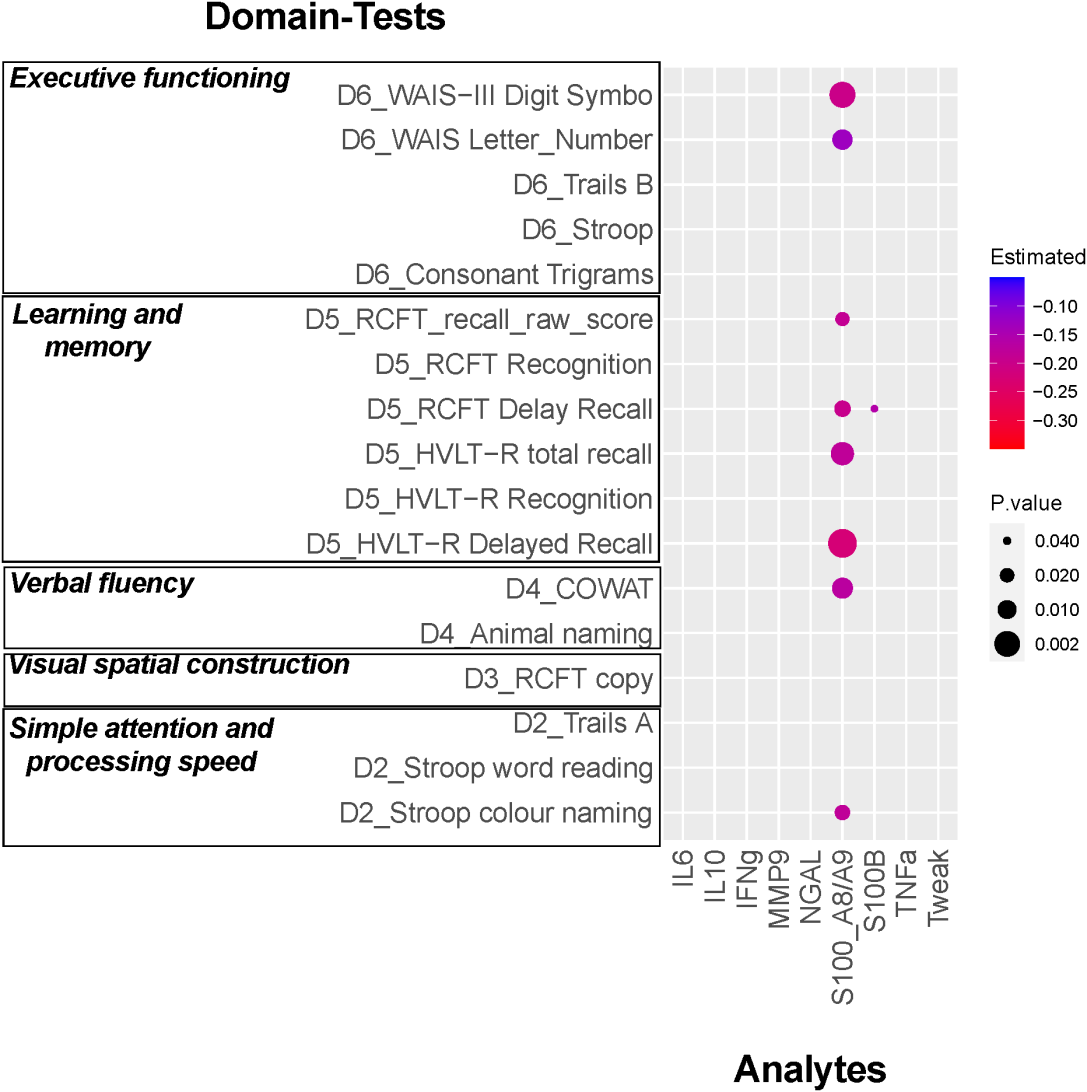
Relationship between the serum analyte levels and each cognitive test Z- score. Results from multivariable analysis controlled by sex, age, race, SLEDAI-2K, SDI, and use of antimalarials, glucocorticoids, biologics, or immunosuppressants. The different analytes values were Log transformed to improve the model fitting. The magnitude of the estimated effect on the reduction in the Z-score by each Log transformed unit of the serum levels of the analyte are indicated by the color and the p-value by the dot size.

**Figure 3** provides a comprehensive summary of the analysis investigating the relationship between serum levels of the analytes and task performance for the various individual tasks that comprise each of the domains. These results highlight the distinct pattern observed for S100A8/A9, which exhibited a negative relationship with multiple CI tests across different domains (Refer to **Supplementary Figure 4** for individual graphs illustrating the relationship between S100A8/A9 and each of the statistically significant tests). These findings contrast with those for all other analytes tested, where a similar relationship with task performance was not seen.

### Analysis of the ability of serum analyte levels to discriminate CI from non-CI in SLE patients

**Figure 4:**
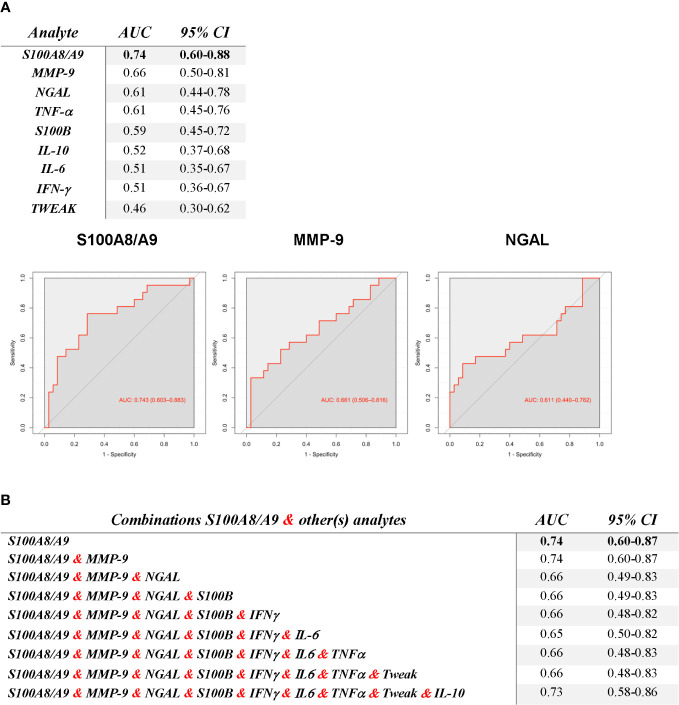
Ability of serum analyte levels to discriminate SLE Patients with Cognitive Impairment (CI) from those without CI, as indicated by the AUC for ROC curves **(A)** and for S100A8/A9 with various combinations of the other analytes **(B)**. The ROC curves of the top 3 analytes are displayed on A.

For this analysis, patients with indeterminate CI were not included. The performance of various analytes in discriminating between SLE patients with and without CI is shown in **Figure 4A**. Notably, S100A8/A9 exhibited the highest AUC (0.74, 95% CI: 0.6-0.88), demonstrating good discriminative ability. The proteases MMP-9 with an AUC of 0. 66 (95% CI: 0.5-0.81) and NGAL with an AUC of 0.61 (95% CI: 0.45-0.77), also demonstrated fair discriminative capabilities. Interestingly, combining serum levels of S100A8/A9 with other analytes did not improve the AUC (**Figure 4B**).

For the three analytes with good or fair discriminative ability, optimal cut-off values to discriminate between CI and non-CI were obtained by Youden’s index. As presented in **Table 2**, S100A8/A9 displayed the greatest discriminative ability (cut-off ≥ 1332.62 ng/mL, resulted in a Sn of 66%, Sp of 66%, PPV of 72%, NPV of 60%, and LR^+^ of 1.96). Refer to **Supplementary Tables 2**, **3** for Sn, Sp, and LR^+^ for each possible S100A8/A9 and MMP-9 cut-off.

**Table 2:** S100A8/A9, MMP-9, NGAL, and various combination’s ability to discriminate SLE patients with CI from those without CI.

Analyte (Cut-off *)	Sn % (95% CI)	Sp % (95% CI)	PPV % (95% CI)	NPV % (95% CI)	LR^+^	LR^-^
S100 A8/A9 (≥ 1332.62 ng/mL)	66 (57-75)	66 (55-76)	72 (62-76)	60 (50-70)	1.96	0.5
MMP-9 (≥ 293.1 ng/mL)	41 (32-50)	75 (65-83)	68 (57-76)	49 (40-62)	1.67	0.77
NGAL (≥ 141.47 pg/mL)	47 (38-56)	70 (60-79)	67 (56-75)	50 (41-62)	1.62	0.74
Analytes Combination	Sn % (95% CI)	Sp % (95% CI)	PPV % (95% CI)	NPV % (95% CI)	LR^+^	LR^-^
S100 A8/A9 and MMP-9	28 (21-37)	84 (75-90)	70 (56-81)	47 (40-55)	1.82	0.85
100 A8/A9 and NGAL	36 (28-45)	83 (74-89)	73 (61-83)	50 (43-58)	2.2	0.77
S100 A8/A9 and MMP-9 and NGAL	23 (16-31)	88 (79-93)	71 (55-83)	47 (40-55)	1.94	0.92

*By Youden’s index.

Sensitivity (Sn), specificity (Sp), positive and negative predictive values (PPV and NPV, respectively), and positive and negative likelihood ratios (LR+ and LR-, respectively).

To explore whether levels above optimal cut-off of these analytes collectively improved discriminatory ability, we assessed the combination of S100A8/A9 with the other two (**Table 2**). While combinations with MMP-9 (≥ 293.1 ng/mL) and NGAL (≥ 141.47 pg/mL) showed improvements in Sp (84%, 83%, respectively), the Sn substantially decreased (less than 30% and 40%, respectively) (**Table 2**).

### Association between the serum levels of S100A8/A9 and inflammatory markers or disease activity

**Supplementary Figure 5** summarizes correlations amongst the different analytes, inflammatory markers (hsCRP and ESR), and disease activity (as measured by the SLEDAI-2K). As can be seen, S100A8/A9 and MMP-9 moderately correlated with each other (ρ=0.52, p<0.0001) and both correlated with NGAL (ρ=0.64, p<0.0001; ρ=0.56, p<0.0001, respectively). Similarly, IFN-γ and S100B had a modest correlation with each other (ρ=0.36, p<0.0001). The serum levels of S100A8/A9 and MMP-9 did not correlate with ESR, hsCRP, disease activity, nor with TNF-α, and there was only a very modest correlation of S100A8/A9 with IL-6 (ρ=0.25, p< =0.0001).

## Discussion

In this study, we investigated differences in serum analyte levels in SLE patients based on their cognitive performance and their potential role in discriminating SLE patients with CI from those without CI. Among the nine analytes measured (IL-6, IL-10, IFN-γ, NGAL, MMP-9, S100A8/A9, S100B, TNF-α, and TWEAK) we found that compared with non-CI SLE patients, those with CI had significantly higher serum levels of S100A8/A9, and to a lesser extent MMP-9.

Overall, our findings are in agreement with a recent cross-sectional study with a smaller sample size (n=72 SLE patients, 26 with CI) that studied the levels of S100A8/A9 in SLE patients with and without NPSLE, where SLE patients with CI had higher serum concentrations when compared with patients without NPSLE (35). However, presumably due to the small sample size, authors did not directly compared CI to non-CI patients.

Notably, S100A8/A9 and MMP-9 have also been implicated in neuroinflammatory processes in other neurological disorders characterized by CI, such as AD and MS (36–41). Overall, these findings provide insight into potential shared molecular pathways contributing to CI across various neurological conditions, thereby enhancing our understanding of the molecular mechanisms underlying CI in SLE. For instance, S100A8/A9, or calprotectin, a heterodimeric protein complex belonging to the S100 family of calcium-binding proteins that is predominantly expressed by neutrophils and monocytes (42), has been implicated in immune cell recruitment to sites of inflammation and activation of the NF-κB pathway, leading to production of proinflammatory cytokines, such as TNF-α and IL-6 (42). In neurological disorders, S100A8/A9 induces reactive oxygen species (ROS) production and microglial activation (36). MMP-9 is involved in extracellular matrix breakdown and BBB remodeling, contributing to peripheral immune cell infiltration into the CNS, activating resident immune cells like microglia (43).

Surprisingly, similar elevations to those seen in SLE patients with CI, were not seen in patients with indeterminate CI. Instead, we noted that serum levels of IFN-γ were elevated in these patients as compared to patients without impairment. This finding suggests that the elevation of this analyte may occur early in the process before the establishment of a definitive cognitive decline. Therefore, IFN-γ might play a role in promoting the dysregulations that lead to the progression and worsening of CI in these patients. In support of this possibility IFN-γ has been shown to upregulate S100A8/A9 (44), which could further enhance neuroinflammation.

Cognitive function is orchestrated by complex interactions between different areas of the brain (45), that to a certain extent are represented by the cognitive domains assessed (46). In our study, we observed that patients with impaired cognitive performance had significantly higher levels of S100A8/A9 across most cognitive domains, while elevated serum levels of IL-6 and TNF-α were only found in patients with impaired simple attention and processing speed. For other analytes, no significant differences in levels were observed when stratified by cognitive domains. These findings suggest distinct roles for these analytes in contributing to CI in SLE patients, with IL-6 and TNF-α affecting specific pathways (*e.g.* front cortical) (47) and S100A8/A9 exerting a more widespread neuroinflammatory effect, impacting multiple brain regions simultaneously (45, 46). In support of this, we found that elevated levels of S100A8/A9 were negatively correlated with multiple cognitive tests across different domains, whereas the other analytes did not show such relationships. Notably, in SLE animal models, IL-6 has been implicated in affecting learning and memory (hippocampal function) (10), however, we did not find increased levels of this cytokine in individuals with impairment in that particular domain. This discrepancy could be attributed to differences in study designs.

Herein we also demonstrated that amongst all measured analytes, S100A8/A9 best differentiated between SLE patients with and without CI, both in terms of AUC and predictive values after establishing cut-off values. In addition to its individual predictive ability, we assessed whether combining S100A8/A9 with the other analytes could improve the discriminative ability. The overall improvement was marginal, however, suggesting that amongst the assessed analytes S100A8/A9 may be the most informative serum analyte for identifying SLE adult patients with CI. Similarly, combining high levels (above cut-off established by Youden index) of S100A8/9 with high levels of MMP-9 and NGAL improved Sp but significantly lowered Sn, suggesting that while S100A8/9, MMP-9, and NGAL may be important in CI development, there are other CI contributors as well. This underscores that CI immunopathogenesis is likely multifactorial, involving multiple pathways and contributing factors.

Interestingly, despite its proinflammatory properties, S100A8/A9 (42) did not correlate with classical markers of systemic inflammation. This agrees with a previous study investigating the role of this cytokine in lupus nephritis (48). One possible explanation proposed by the authors is the known low accuracy of these markers in SLE. However, we also found that S100A8/A9 did not correlate with other surrogate markers of systemic inflammation, such as IL-6, TNF-α, or the SLEDAI-2K. It is worth noting that our study population had low disease activity, which could account for the lack of correlation with disease activity measures.

One of the key strengths of our study is the large number of participants, which allowed for robust conclusions to be drawn from the data. We also investigated the potential confounding effects of medications on analyte levels, which adds further validity to our findings. A key point in our study is its clear focus on CI. However, since our study was cross-sectional, we cannot establish causality. To address this limitation, analysis of longitudinal data is required. Our study’s primary aim was not to establish whether these analytes are elevated in SLE patients compared to individuals with other rheumatic diseases or healthy controls. Instead, the focus was on exploring differences in analytes levels within the group of SLE patients based on their cognitive performance independently from the attribution to SLE. While future research may indeed benefit from including control groups to assess biomarker specificity, the absence of such groups should not detract from the study’s ability to shed light on the relationship between S100A8/A9 and MMP-9 and CI within the context of SLE. Another potential limitation of our study is that we only measured nine analytes, however, the selected analytes were derived from the literature, and we believe they are highly representative. We should also consider that cytokines were measured in serum instead of plasma. However, our evaluation of storage effect on serum levels showed no significant impact. Additionally, all sample concentrations were based on the same matrix (serum). Thus, if there were any stability issues, they would be consistent across all participants. Finally, we only measured analytes in serum and did not assess their levels in CSF, and a previous work that included 30 SLE patients with CNS NPSLE, yet no CI, showed that for some analytes (including IL-6, IL-10, IFN-γ, and TNFα) the serum levels did not correlate with the CSF levels (49), therefore it remains possible that some of the measured analytes are produced locally in the brain and that their elevated levels are not reflected in the circulation. Consequently, our study design cannot rule out their contribution to local neuroinflammation. However, it is important to consider that lumbar puncture is a more invasive procedure and may not be indicated or feasible for the majority of patients. In contrast, the identification of serum biomarkers offers a more convenient and accessible approach for clinical use. Despite the limitations in assessing local neuroinflammation, serum biomarkers still provide important information and can serve as valuable indicators for disease assessment and management. Future research may further investigate the relationship between serum and CSF levels of S100A8/A9 and MMP-9 to gain a more comprehensive understanding of their roles in the pathogenesis of CI in SLE. Likewise, we acknowledge that although there were statistically significant variations in serum levels of S100A8/9 and MMP-9 between individuals without CI and those with CI, the practical utility of these analytes as clinical biomarkers may be limited due to their relatively low Sp and Sn. Similarly, an ideal clinical biomarker should demonstrate better PPV and NPV. Nevertheless, these significant associations, particularly for S100 A8/9 may suggest potential pathways worth exploring in future research.

## Conclusion

In conclusion, our study underscores the possible involvement of S100A8/A9 and MMP-9 in the immunopathogenesis of CI in adult SLE patients. Our results contribute to a deeper understanding of CI in the context of SLE and provide valuable insights into potential therapeutic approaches for mitigating cognitive dysfunction in this patient population. Nonetheless, further research is essential to elucidate the precise mechanisms underlying our observations and to validate the role of S100A8/A9 and MMP-9 in CI in the context of SLE.

## Data availability statement

The original contributions presented in the study are included in the article/**Supplementary Material**. Further inquiries can be directed to the corresponding author.

## Ethics statement

The studies involving humans were approved by Research Ethics Board of the University Health Network (REB# 15-9582). The studies were conducted in accordance with the local legislation and institutional requirements. The participants provided their written informed consent to participate in this study.

## Author contributions

CM: Writing – original draft, Writing – review & editing. MB: Writing – original draft, Writing – review & editing. JD: Writing – original draft, Writing – review & editing. JS: Writing – original draft, Writing – review & editing. KB: Writing – original draft, Writing – review & editing. MK: Writing – original draft, Writing – review & editing. RK: Writing – original draft, Writing – review & editing. MT: Writing – original draft, Writing – review & editing. LR: Writing – original draft, Writing – review & editing. MC: Writing – original draft, Writing – review & editing. SA: Writing – original draft, Writing – review & editing. SM: Writing – original draft, Writing – review & editing. DEB: Writing – original draft, Writing – review & editing. PK: Writing – original draft, Writing – review & editing. DOB: Writing – original draft, Writing – review & editing. RG: Writing – original draft, Writing – review & editing. DG: Writing – original draft, Writing – review & editing. JW: Writing – original draft, Writing – review & editing. ZT: Writing – original draft, Writing – review & editing.
