# Supplementary material for: Serum S100A8/A9 and MMP-9 levels are elevated in systemic lupus erythematosus patients with cognitive impairment
Source: Front Immunol. 2024 Jan 25;14:1326751. doi: 10.3389/fimmu.2023.1326751 (PMC10851148; doi:10.3389/fimmu.2023.1326751)
Supplement: Supplementary file 1 [file DataSheet_1.docx]

**Supplementary Table 1.**

**Measured analytes ELISA parameters (dilution, dynamic range, and sensitivity)**

| **Analyte** | **Dilution** | **Dynamic range** | **Sensitivity** |
| --- | --- | --- | --- |
| **DuoSet ELISA** |  |  |  |
| S100A8/A9 | 1: 2000 | 94-6000 pg/mL |  |
| NGAL | 1: 1000 | 78.1-5000 pg/mL |  |
| MMP-9 | 1: 1500 | 31.2-2000 pg/mL |  |
| S100B | 1: 2 | 46.9-3000 pg/mL |  |
| TWEAK | 1: 2 | 62.5-4000 pg/mL |  |
| **hsELISA** |  |  |  |
| TNF-α | Neat | 15.6-1000 pg/mL | 6.23 pg/mL |
| IL-6 | Neat | 3.1-300 pg/mL | 0.7 pg/mL |
| IFN-γ | Neat | 0.16-10 pg/mL | 0.06 pg/mL |
| IL-10 | Neat | 7.8-500 pg/mL | 3.9 pg/mL |

*High sensitivity (hs)*

**Supplementary Table 2. Different S100A8/A9 Cut-off’s diagnostic accuracy measures for CI**

| Cut-off | Sn % | 95% CI | Sp % | 95% CI | LR+ |
| --- | --- | --- | --- | --- | --- |
| > 66.57 | 100.0 | 96.79% to 100.0% | 1.124 | 0.05763% to 6.093% | 1.011 |
| > 118.9 | 99.14 | 95.28% to 99.96% | 1.124 | 0.05763% to 6.093% | 1.003 |
| > 135.1 | 99.14 | 95.28% to 99.96% | 2.247 | 0.3993% to 7.828% | 1.014 |
| > 146.3 | 98.28 | 93.93% to 99.69% | 2.247 | 0.3993% to 7.828% | 1.005 |
| > 152.4 | 98.28 | 93.93% to 99.69% | 3.371 | 0.9188% to 9.447% | 1.017 |
| > 161.2 | 97.41 | 92.67% to 99.30% | 3.371 | 0.9188% to 9.447% | 1.008 |
| > 182.5 | 97.41 | 92.67% to 99.30% | 4.494 | 1.761% to 10.99% | 1.020 |
| > 211.4 | 97.41 | 92.67% to 99.30% | 5.618 | 2.423% to 12.49% | 1.032 |
| > 227.4 | 97.41 | 92.67% to 99.30% | 6.742 | 3.126% to 13.94% | 1.045 |
| > 230.5 | 97.41 | 92.67% to 99.30% | 7.865 | 3.862% to 15.36% | 1.057 |
| > 244.0 | 97.41 | 92.67% to 99.30% | 8.989 | 4.625% to 16.75% | 1.070 |
| > 259.3 | 97.41 | 92.67% to 99.30% | 10.11 | 5.412% to 18.11% | 1.084 |
| > 264.6 | 96.55 | 91.47% to 98.65% | 10.11 | 5.412% to 18.11% | 1.074 |
| > 266.8 | 96.55 | 91.47% to 98.65% | 11.24 | 6.219% to 19.46% | 1.088 |
| > 281.2 | 96.55 | 91.47% to 98.65% | 12.36 | 7.044% to 20.79% | 1.102 |
| > 298.8 | 96.55 | 91.47% to 98.65% | 13.48 | 7.884% to 22.10% | 1.116 |
| > 304.2 | 96.55 | 91.47% to 98.65% | 14.61 | 8.739% to 23.40% | 1.131 |
| > 307.3 | 96.55 | 91.47% to 98.65% | 15.73 | 9.608% to 24.69% | 1.146 |
| > 309.9 | 95.69 | 90.31% to 98.15% | 15.73 | 9.608% to 24.69% | 1.136 |
| > 315.0 | 95.69 | 90.31% to 98.15% | 16.85 | 10.49% to 25.96% | 1.151 |
| > 320.0 | 95.69 | 90.31% to 98.15% | 17.98 | 11.38% to 27.23% | 1.167 |
| > 328.8 | 95.69 | 90.31% to 98.15% | 19.10 | 12.28% to 28.48% | 1.183 |
| > 337.3 | 95.69 | 90.31% to 98.15% | 20.22 | 13.19% to 29.72% | 1.199 |
| > 338.5 | 95.69 | 90.31% to 98.15% | 21.35 | 14.11% to 30.95% | 1.217 |
| > 345.4 | 95.69 | 90.31% to 98.15% | 22.47 | 15.04% to 32.18% | 1.234 |
| > 370.2 | 95.69 | 90.31% to 98.15% | 23.60 | 15.98% to 33.39% | 1.252 |
| > 392.6 | 94.83 | 89.17% to 97.61% | 23.60 | 15.98% to 33.39% | 1.241 |
| > 397.1 | 93.97 | 88.07% to 97.05% | 23.60 | 15.98% to 33.39% | 1.230 |
| > 404.4 | 93.10 | 86.98% to 96.46% | 23.60 | 15.98% to 33.39% | 1.219 |
| > 412.0 | 92.24 | 85.91% to 95.86% | 23.60 | 15.98% to 33.39% | 1.207 |
| > 445.0 | 92.24 | 85.91% to 95.86% | 24.72 | 16.93% to 34.60% | 1.225 |
| > 479.4 | 92.24 | 85.91% to 95.86% | 25.84 | 17.88% to 35.80% | 1.244 |
| > 481.9 | 91.38 | 84.86% to 95.25% | 25.84 | 17.88% to 35.80% | 1.232 |
| > 489.3 | 90.52 | 83.81% to 94.62% | 25.84 | 17.88% to 35.80% | 1.221 |
| > 497.1 | 89.66 | 82.79% to 93.98% | 25.84 | 17.88% to 35.80% | 1.209 |
| > 515.6 | 89.66 | 82.79% to 93.98% | 26.97 | 18.84% to 37.00% | 1.228 |
| > 539.7 | 88.79 | 81.77% to 93.33% | 26.97 | 18.84% to 37.00% | 1.216 |
| > 546.6 | 87.93 | 80.76% to 92.67% | 26.97 | 18.84% to 37.00% | 1.204 |
| > 547.2 | 87.07 | 79.76% to 92.00% | 26.97 | 18.84% to 37.00% | 1.192 |
| > 552.3 | 86.21 | 78.76% to 91.33% | 26.97 | 18.84% to 37.00% | 1.180 |
| > 560.7 | 86.21 | 78.76% to 91.33% | 28.09 | 19.81% to 38.18% | 1.199 |
| > 574.2 | 85.34 | 77.78% to 90.64% | 28.09 | 19.81% to 38.18% | 1.187 |
| > 593.9 | 85.34 | 77.78% to 90.64% | 29.21 | 20.78% to 39.36% | 1.206 |
| > 615.5 | 85.34 | 77.78% to 90.64% | 30.34 | 21.76% to 40.54% | 1.225 |
| > 630.0 | 84.48 | 76.80% to 89.95% | 30.34 | 21.76% to 40.54% | 1.213 |
| > 637.4 | 84.48 | 76.80% to 89.95% | 31.46 | 22.75% to 41.70% | 1.233 |
| > 671.6 | 83.62 | 75.83% to 89.26% | 31.46 | 22.75% to 41.70% | 1.220 |
| > 701.2 | 82.76 | 74.86% to 88.55% | 31.46 | 22.75% to 41.70% | 1.207 |
| > 707.7 | 82.76 | 74.86% to 88.55% | 32.58 | 23.74% to 42.87% | 1.228 |
| > 716.6 | 82.76 | 74.86% to 88.55% | 33.71 | 24.74% to 44.02% | 1.248 |
| > 720.8 | 81.90 | 73.90% to 87.84% | 33.71 | 24.74% to 44.02% | 1.235 |
| > 723.3 | 81.03 | 72.95% to 87.13% | 33.71 | 24.74% to 44.02% | 1.222 |
| > 726.2 | 81.03 | 72.95% to 87.13% | 34.83 | 25.75% to 45.17% | 1.243 |
| > 732.8 | 81.03 | 72.95% to 87.13% | 35.96 | 26.76% to 46.31% | 1.265 |
| > 741.1 | 81.03 | 72.95% to 87.13% | 37.08 | 27.77% to 47.45% | 1.288 |
| > 746.5 | 81.03 | 72.95% to 87.13% | 38.20 | 28.79% to 48.59% | 1.311 |
| > 751.4 | 81.03 | 72.95% to 87.13% | 39.33 | 29.82% to 49.71% | 1.336 |
| > 757.7 | 81.03 | 72.95% to 87.13% | 40.45 | 30.85% to 50.84% | 1.361 |
| > 765.8 | 80.17 | 72.00% to 86.41% | 40.45 | 30.85% to 50.84% | 1.346 |
| > 780.8 | 80.17 | 72.00% to 86.41% | 41.57 | 31.89% to 51.95% | 1.372 |
| > 801.8 | 79.31 | 71.06% to 85.68% | 41.57 | 31.89% to 51.95% | 1.357 |
| > 817.2 | 78.45 | 70.12% to 84.95% | 41.57 | 31.89% to 51.95% | 1.343 |
| > 823.1 | 77.59 | 69.18% to 84.22% | 41.57 | 31.89% to 51.95% | 1.328 |
| > 827.8 | 77.59 | 69.18% to 84.22% | 42.70 | 32.93% to 53.06% | 1.354 |
| > 838.9 | 75.86 | 67.33% to 82.74% | 42.70 | 32.93% to 53.06% | 1.324 |
| > 857.7 | 75.86 | 67.33% to 82.74% | 43.82 | 33.98% to 54.17% | 1.350 |
| > 870.4 | 75.86 | 67.33% to 82.74% | 44.94 | 35.03% to 55.27% | 1.378 |
| > 881.8 | 75.86 | 67.33% to 82.74% | 46.07 | 36.09% to 56.37% | 1.407 |
| > 894.7 | 75.86 | 67.33% to 82.74% | 47.19 | 37.15% to 57.46% | 1.437 |
| > 917.0 | 75.86 | 67.33% to 82.74% | 48.31 | 38.22% to 58.55% | 1.468 |
| > 942.4 | 75.86 | 67.33% to 82.74% | 49.44 | 39.29% to 59.63% | 1.500 |
| > 956.1 | 75.86 | 67.33% to 82.74% | 50.56 | 40.37% to 60.71% | 1.534 |
| > 966.8 | 75.00 | 66.40% to 81.99% | 50.56 | 40.37% to 60.71% | 1.517 |
| > 983.5 | 74.14 | 65.49% to 81.24% | 50.56 | 40.37% to 60.71% | 1.500 |
| > 998.2 | 74.14 | 65.49% to 81.24% | 51.69 | 41.45% to 61.78% | 1.534 |
| > 1006 | 73.28 | 64.57% to 80.49% | 51.69 | 41.45% to 61.78% | 1.517 |
| > 1017 | 72.41 | 63.66% to 79.73% | 51.69 | 41.45% to 61.78% | 1.499 |
| > 1029 | 72.41 | 63.66% to 79.73% | 52.81 | 42.54% to 62.85% | 1.534 |
| > 1044 | 71.55 | 62.75% to 78.97% | 52.81 | 42.54% to 62.85% | 1.516 |
| > 1053 | 70.69 | 61.85% to 78.20% | 52.81 | 42.54% to 62.85% | 1.498 |
| > 1056 | 70.69 | 61.85% to 78.20% | 53.93 | 43.63% to 63.91% | 1.534 |
| > 1085 | 69.83 | 60.95% to 77.43% | 53.93 | 43.63% to 63.91% | 1.516 |
| > 1114 | 69.83 | 60.95% to 77.43% | 55.06 | 44.73% to 64.97% | 1.554 |
| > 1123 | 68.97 | 60.05% to 76.66% | 55.06 | 44.73% to 64.97% | 1.534 |
| > 1134 | 68.97 | 60.05% to 76.66% | 56.18 | 45.83% to 66.02% | 1.574 |
| > 1146 | 68.10 | 59.16% to 75.89% | 56.18 | 45.83% to 66.02% | 1.554 |
| > 1167 | 68.10 | 59.16% to 75.89% | 57.30 | 46.94% to 67.07% | 1.595 |
| > 1188 | 67.24 | 58.27% to 75.11% | 57.30 | 46.94% to 67.07% | 1.575 |
| > 1211 | 67.24 | 58.27% to 75.11% | 58.43 | 48.05% to 68.11% | 1.617 |
| > 1229 | 67.24 | 58.27% to 75.11% | 59.55 | 49.16% to 69.15% | 1.662 |
| > 1240 | 66.38 | 57.38% to 74.33% | 59.55 | 49.16% to 69.15% | 1.641 |
| > 1267 | 66.38 | 57.38% to 74.33% | 60.67 | 50.29% to 70.18% | 1.688 |
| > 1289 | 66.38 | 57.38% to 74.33% | 61.80 | 51.41% to 71.21% | 1.738 |
| > 1303 | 66.38 | 57.38% to 74.33% | 62.92 | 52.55% to 72.23% | 1.790 |
| > 1316 | 66.38 | 57.38% to 74.33% | 64.04 | 53.69% to 73.24% | 1.846 |
| > 1324 | 66.38 | 57.38% to 74.33% | 65.17 | 54.83% to 74.25% | 1.906 |
| * > 1333 | 66.38 | 57.38% to 74.33% | 66.29 | 55.98% to 75.26% | 1.969 |
| > 1334 | 64.66 | 55.61% to 72.76% | 66.29 | 55.98% to 75.26% | 1.918 |
| > 1341 | 63.79 | 54.74% to 71.97% | 66.29 | 55.98% to 75.26% | 1.893 |
| > 1365 | 63.79 | 54.74% to 71.97% | 67.42 | 57.13% to 76.26% | 1.958 |
| > 1386 | 63.79 | 54.74% to 71.97% | 68.54 | 58.30% to 77.25% | 2.028 |
| > 1419 | 62.93 | 53.86% to 71.17% | 68.54 | 58.30% to 77.25% | 2.000 |
| > 1451 | 62.07 | 52.99% to 70.38% | 68.54 | 58.30% to 77.25% | 1.973 |
| > 1456 | 61.21 | 52.12% to 69.58% | 68.54 | 58.30% to 77.25% | 1.946 |
| > 1460 | 60.34 | 51.25% to 68.78% | 68.54 | 58.30% to 77.25% | 1.918 |
| > 1469 | 59.48 | 50.38% to 67.97% | 68.54 | 58.30% to 77.25% | 1.891 |
| > 1482 | 58.62 | 49.52% to 67.17% | 68.54 | 58.30% to 77.25% | 1.863 |
| > 1496 | 57.76 | 48.66% to 66.36% | 68.54 | 58.30% to 77.25% | 1.836 |
| > 1510 | 57.76 | 48.66% to 66.36% | 69.66 | 59.46% to 78.24% | 1.904 |
| > 1518 | 56.90 | 47.81% to 65.54% | 69.66 | 59.46% to 78.24% | 1.875 |
| > 1559 | 56.90 | 47.81% to 65.54% | 70.79 | 60.64% to 79.22% | 1.948 |
| > 1599 | 56.03 | 46.95% to 64.73% | 70.79 | 60.64% to 79.22% | 1.918 |
| > 1605 | 56.03 | 46.95% to 64.73% | 71.91 | 61.82% to 80.19% | 1.995 |
| > 1627 | 55.17 | 46.10% to 63.91% | 71.91 | 61.82% to 80.19% | 1.964 |
| > 1645 | 54.31 | 45.25% to 63.09% | 71.91 | 61.82% to 80.19% | 1.933 |
| > 1651 | 54.31 | 45.25% to 63.09% | 73.03 | 63.00% to 81.16% | 2.014 |
| > 1671 | 53.45 | 44.41% to 62.27% | 73.03 | 63.00% to 81.16% | 1.982 |
| > 1698 | 53.45 | 44.41% to 62.27% | 74.16 | 64.20% to 82.12% | 2.068 |
| > 1712 | 53.45 | 44.41% to 62.27% | 75.28 | 65.40% to 83.07% | 2.162 |
| > 1719 | 53.45 | 44.41% to 62.27% | 76.40 | 66.61% to 84.02% | 2.265 |
| > 1726 | 52.59 | 43.56% to 61.44% | 76.40 | 66.61% to 84.02% | 2.229 |
| > 1733 | 51.72 | 42.72% to 60.62% | 76.40 | 66.61% to 84.02% | 2.192 |
| > 1742 | 50.86 | 41.88% to 59.79% | 76.40 | 66.61% to 84.02% | 2.156 |
| > 1756 | 50.86 | 41.88% to 59.79% | 77.53 | 67.82% to 84.96% | 2.263 |
| > 1802 | 50.00 | 41.05% to 58.95% | 77.53 | 67.82% to 84.96% | 2.225 |
| > 1841 | 50.00 | 41.05% to 58.95% | 78.65 | 69.05% to 85.89% | 2.342 |
| > 1846 | 49.14 | 40.21% to 58.12% | 78.65 | 69.05% to 85.89% | 2.302 |
| > 1860 | 48.28 | 39.38% to 57.28% | 78.65 | 69.05% to 85.89% | 2.261 |
| > 1873 | 47.41 | 38.56% to 56.44% | 78.65 | 69.05% to 85.89% | 2.221 |
| > 1898 | 47.41 | 38.56% to 56.44% | 79.78 | 70.28% to 86.81% | 2.344 |
| > 1931 | 47.41 | 38.56% to 56.44% | 80.90 | 71.52% to 87.72% | 2.482 |
| > 1949 | 46.55 | 37.73% to 55.59% | 80.90 | 71.52% to 87.72% | 2.437 |
| > 1962 | 46.55 | 37.73% to 55.59% | 82.02 | 72.77% to 88.62% | 2.589 |
| > 1968 | 46.55 | 37.73% to 55.59% | 83.15 | 74.04% to 89.51% | 2.762 |
| > 1980 | 45.69 | 36.91% to 54.75% | 83.15 | 74.04% to 89.51% | 2.711 |
| > 1996 | 44.83 | 36.09% to 53.90% | 83.15 | 74.04% to 89.51% | 2.660 |
| > 2008 | 44.83 | 36.09% to 53.90% | 84.27 | 75.31% to 90.39% | 2.850 |
| > 2028 | 43.97 | 35.27% to 53.05% | 84.27 | 75.31% to 90.39% | 2.795 |
| > 2050 | 43.10 | 34.46% to 52.19% | 84.27 | 75.31% to 90.39% | 2.740 |
| > 2073 | 43.10 | 34.46% to 52.19% | 85.39 | 76.60% to 91.26% | 2.951 |
| > 2158 | 42.24 | 33.64% to 51.34% | 85.39 | 76.60% to 91.26% | 2.892 |
| > 2252 | 42.24 | 33.64% to 51.34% | 86.52 | 77.90% to 92.12% | 3.133 |
| > 2275 | 41.38 | 32.83% to 50.48% | 86.52 | 77.90% to 92.12% | 3.069 |
| > 2284 | 41.38 | 32.83% to 50.48% | 87.64 | 79.21% to 92.96% | 3.348 |
| > 2298 | 41.38 | 32.83% to 50.48% | 88.76 | 80.54% to 93.78% | 3.683 |
| > 2309 | 40.52 | 32.03% to 49.62% | 88.76 | 80.54% to 93.78% | 3.606 |
| > 2318 | 39.66 | 31.22% to 48.75% | 88.76 | 80.54% to 93.78% | 3.529 |
| > 2331 | 38.79 | 30.42% to 47.88% | 88.76 | 80.54% to 93.78% | 3.453 |
| > 2354 | 38.79 | 30.42% to 47.88% | 89.89 | 81.89% to 94.59% | 3.836 |
| > 2398 | 37.93 | 29.62% to 47.01% | 89.89 | 81.89% to 94.59% | 3.751 |
| > 2428 | 37.07 | 28.83% to 46.14% | 89.89 | 81.89% to 94.59% | 3.666 |
| > 2445 | 36.21 | 28.03% to 45.26% | 89.89 | 81.89% to 94.59% | 3.580 |
| > 2490 | 35.34 | 27.24% to 44.39% | 89.89 | 81.89% to 94.59% | 3.495 |
| > 2563 | 34.48 | 26.46% to 43.50% | 89.89 | 81.89% to 94.59% | 3.410 |
| > 2608 | 33.62 | 25.67% to 42.62% | 89.89 | 81.89% to 94.59% | 3.325 |
| > 2656 | 32.76 | 24.89% to 41.73% | 89.89 | 81.89% to 94.59% | 3.239 |
| > 2728 | 32.76 | 24.89% to 41.73% | 91.01 | 83.25% to 95.37% | 3.644 |
| > 2756 | 31.90 | 24.11% to 40.84% | 91.01 | 83.25% to 95.37% | 3.548 |
| > 2784 | 31.03 | 23.34% to 39.95% | 91.01 | 83.25% to 95.37% | 3.453 |
| > 2817 | 30.17 | 22.57% to 39.05% | 91.01 | 83.25% to 95.37% | 3.357 |
| > 2868 | 29.31 | 21.80% to 38.15% | 91.01 | 83.25% to 95.37% | 3.261 |
| > 2947 | 29.31 | 21.80% to 38.15% | 92.13 | 84.64% to 96.14% | 3.727 |
| > 3061 | 28.45 | 21.03% to 37.25% | 92.13 | 84.64% to 96.14% | 3.617 |
| > 3174 | 28.45 | 21.03% to 37.25% | 93.26 | 86.06% to 96.87% | 4.220 |
| > 3225 | 28.45 | 21.03% to 37.25% | 94.38 | 87.51% to 97.58% | 5.064 |
| > 3245 | 27.59 | 20.27% to 36.34% | 94.38 | 87.51% to 97.58% | 4.910 |
| > 3266 | 26.72 | 19.51% to 35.43% | 94.38 | 87.51% to 97.58% | 4.757 |
| > 3317 | 26.72 | 19.51% to 35.43% | 95.51 | 89.01% to 98.24% | 5.946 |
| > 3354 | 25.86 | 18.76% to 34.51% | 95.51 | 89.01% to 98.24% | 5.754 |
| > 3456 | 25.00 | 18.01% to 33.60% | 95.51 | 89.01% to 98.24% | 5.563 |
| > 3558 | 24.14 | 17.26% to 32.67% | 95.51 | 89.01% to 98.24% | 5.371 |
| > 3581 | 23.28 | 16.52% to 31.75% | 95.51 | 89.01% to 98.24% | 5.179 |
| > 3602 | 22.41 | 15.78% to 30.82% | 95.51 | 89.01% to 98.24% | 4.987 |
| > 3620 | 22.41 | 15.78% to 30.82% | 96.63 | 90.55% to 99.08% | 6.649 |
| > 3664 | 21.55 | 15.05% to 29.88% | 96.63 | 90.55% to 99.08% | 6.394 |
| > 3732 | 20.69 | 14.32% to 28.94% | 96.63 | 90.55% to 99.08% | 6.138 |
| > 3789 | 19.83 | 13.59% to 28.00% | 96.63 | 90.55% to 99.08% | 5.882 |
| > 3834 | 18.97 | 12.87% to 27.05% | 96.63 | 90.55% to 99.08% | 5.626 |
| > 3930 | 18.10 | 12.16% to 26.10% | 96.63 | 90.55% to 99.08% | 5.371 |
| > 4026 | 17.24 | 11.45% to 25.14% | 96.63 | 90.55% to 99.08% | 5.115 |
| > 4072 | 16.38 | 10.74% to 24.17% | 96.63 | 90.55% to 99.08% | 4.859 |
| > 4107 | 15.52 | 10.05% to 23.20% | 96.63 | 90.55% to 99.08% | 4.603 |
| > 4153 | 14.66 | 9.356% to 22.22% | 96.63 | 90.55% to 99.08% | 4.348 |
| > 4189 | 14.66 | 9.356% to 22.22% | 97.75 | 92.17% to 99.60% | 6.522 |
| > 4237 | 13.79 | 8.672% to 21.24% | 97.75 | 92.17% to 99.60% | 6.138 |
| > 4400 | 12.93 | 7.995% to 20.24% | 97.75 | 92.17% to 99.60% | 5.754 |
| > 4657 | 12.93 | 7.995% to 20.24% | 98.88 | 93.91% to 99.94% | 11.51 |
| > 4871 | 12.07 | 7.327% to 19.24% | 98.88 | 93.91% to 99.94% | 10.74 |
| > 5029 | 11.21 | 6.667% to 18.23% | 98.88 | 93.91% to 99.94% | 9.974 |
| > 5180 | 10.34 | 6.017% to 17.21% | 98.88 | 93.91% to 99.94% | 9.207 |
| > 5257 | 9.483 | 5.378% to 16.19% | 98.88 | 93.91% to 99.94% | 8.440 |
| > 5286 | 8.621 | 4.750% to 15.14% | 98.88 | 93.91% to 99.94% | 7.672 |
| > 5430 | 8.621 | 4.750% to 15.14% | 100.0 | 95.86% to 100.0% |  |

**Youden’s index cut-off*

*Sensitivity (Sn), specificity (Sp), positive and negative predictive values (PPV and NPV,*

*respectively), and positive likelihood ratis (LR+)*

**Supplementary Table 3. Different MMP-9 Cut-off’s diagnostic accuracy measures for CI**

| Cut-off | Sn % | 95% CI | Sp % | 95% CI | LR+ |
| --- | --- | --- | --- | --- | --- |
| > 9.819 | 100.0 | 96.79% to 100.0% | 1.099 | 0.05637% to 5.965% | 1.011 |
| > 16.12 | 100.0 | 96.79% to 100.0% | 2.198 | 0.3905% to 7.663% | 1.022 |
| > 18.62 | 100.0 | 96.79% to 100.0% | 3.297 | 0.8986% to 9.249% | 1.034 |
| > 20.45 | 99.14 | 95.28% to 99.96% | 3.297 | 0.8986% to 9.249% | 1.025 |
| > 21.03 | 98.28 | 93.93% to 99.69% | 3.297 | 0.8986% to 9.249% | 1.016 |
| > 22.71 | 97.41 | 92.67% to 99.30% | 3.297 | 0.8986% to 9.249% | 1.007 |
| > 24.28 | 97.41 | 92.67% to 99.30% | 4.396 | 1.722% to 10.76% | 1.019 |
| > 25.74 | 96.55 | 91.47% to 98.65% | 4.396 | 1.722% to 10.76% | 1.010 |
| > 27.06 | 96.55 | 91.47% to 98.65% | 5.495 | 2.370% to 12.22% | 1.022 |
| > 27.47 | 95.69 | 90.31% to 98.15% | 5.495 | 2.370% to 12.22% | 1.013 |
| > 27.73 | 95.69 | 90.31% to 98.15% | 6.593 | 3.057% to 13.65% | 1.024 |
| > 27.91 | 95.69 | 90.31% to 98.15% | 7.692 | 3.776% to 15.04% | 1.037 |
| > 29.17 | 94.83 | 89.17% to 97.61% | 7.692 | 3.776% to 15.04% | 1.027 |
| > 31.14 | 93.97 | 88.07% to 97.05% | 7.692 | 3.776% to 15.04% | 1.018 |
| > 31.99 | 93.97 | 88.07% to 97.05% | 8.791 | 4.522% to 16.40% | 1.030 |
| > 32.12 | 93.97 | 88.07% to 97.05% | 9.890 | 5.291% to 17.74% | 1.043 |
| > 32.25 | 93.97 | 88.07% to 97.05% | 10.99 | 6.079% to 19.06% | 1.056 |
| > 33.44 | 93.10 | 86.98% to 96.46% | 10.99 | 6.079% to 19.06% | 1.046 |
| > 35.02 | 92.24 | 85.91% to 95.86% | 10.99 | 6.079% to 19.06% | 1.036 |
| > 36.82 | 91.38 | 84.86% to 95.25% | 10.99 | 6.079% to 19.06% | 1.027 |
| > 38.57 | 90.52 | 83.81% to 94.62% | 10.99 | 6.079% to 19.06% | 1.017 |
| > 38.99 | 90.52 | 83.81% to 94.62% | 12.09 | 6.886% to 20.36% | 1.030 |
| > 39.44 | 89.66 | 82.79% to 93.98% | 12.09 | 6.886% to 20.36% | 1.020 |
| > 40.01 | 89.66 | 82.79% to 93.98% | 13.19 | 7.707% to 21.65% | 1.033 |
| > 41.14 | 89.66 | 82.79% to 93.98% | 14.29 | 8.543% to 22.92% | 1.046 |
| > 42.71 | 89.66 | 82.79% to 93.98% | 15.38 | 9.391% to 24.18% | 1.060 |
| > 43.38 | 89.66 | 82.79% to 93.98% | 16.48 | 10.25% to 25.43% | 1.074 |
| > 43.59 | 89.66 | 82.79% to 93.98% | 17.58 | 11.12% to 26.67% | 1.088 |
| > 44.54 | 88.79 | 81.77% to 93.33% | 17.58 | 11.12% to 26.67% | 1.077 |
| > 45.34 | 88.79 | 81.77% to 93.33% | 18.68 | 12.00% to 27.90% | 1.092 |
| > 45.76 | 88.79 | 81.77% to 93.33% | 19.78 | 12.89% to 29.11% | 1.107 |
| > 47.65 | 87.93 | 80.76% to 92.67% | 19.78 | 12.89% to 29.11% | 1.096 |
| > 49.51 | 87.07 | 79.76% to 92.00% | 19.78 | 12.89% to 29.11% | 1.085 |
| > 50.56 | 86.21 | 78.76% to 91.33% | 20.88 | 13.79% to 30.32% | 1.090 |
| > 51.55 | 86.21 | 78.76% to 91.33% | 21.98 | 14.70% to 31.52% | 1.105 |
| > 51.91 | 86.21 | 78.76% to 91.33% | 23.08 | 15.62% to 32.72% | 1.121 |
| > 53.59 | 85.34 | 77.78% to 90.64% | 23.08 | 15.62% to 32.72% | 1.109 |
| > 55.25 | 85.34 | 77.78% to 90.64% | 24.18 | 16.54% to 33.90% | 1.126 |
| > 55.34 | 84.48 | 76.80% to 89.95% | 24.18 | 16.54% to 33.90% | 1.114 |
| > 55.57 | 83.62 | 75.83% to 89.26% | 24.18 | 16.54% to 33.90% | 1.103 |
| > 56.23 | 82.76 | 74.86% to 88.55% | 24.18 | 16.54% to 33.90% | 1.091 |
| > 56.91 | 81.90 | 73.90% to 87.84% | 24.18 | 16.54% to 33.90% | 1.080 |
| > 57.77 | 81.03 | 72.95% to 87.13% | 25.27 | 17.47% to 35.08% | 1.084 |
| > 58.81 | 80.17 | 72.00% to 86.41% | 25.27 | 17.47% to 35.08% | 1.073 |
| > 59.16 | 79.31 | 71.06% to 85.68% | 25.27 | 17.47% to 35.08% | 1.061 |
| > 61.60 | 78.45 | 70.12% to 84.95% | 25.27 | 17.47% to 35.08% | 1.050 |
| > 64.20 | 78.45 | 70.12% to 84.95% | 26.37 | 18.41% to 36.25% | 1.065 |
| > 66.05 | 78.45 | 70.12% to 84.95% | 27.47 | 19.36% to 37.41% | 1.082 |
| > 69.03 | 78.45 | 70.12% to 84.95% | 28.57 | 20.31% to 38.57% | 1.098 |
| > 70.36 | 77.59 | 69.18% to 84.22% | 28.57 | 20.31% to 38.57% | 1.086 |
| > 70.74 | 77.59 | 69.18% to 84.22% | 29.67 | 21.26% to 39.72% | 1.103 |
| > 73.38 | 77.59 | 69.18% to 84.22% | 30.77 | 22.23% to 40.87% | 1.121 |
| > 76.07 | 77.59 | 69.18% to 84.22% | 31.87 | 23.20% to 42.01% | 1.139 |
| > 77.46 | 77.59 | 69.18% to 84.22% | 32.97 | 24.17% to 43.14% | 1.157 |
| > 78.49 | 76.72 | 68.25% to 83.48% | 32.97 | 24.17% to 43.14% | 1.145 |
| > 80.63 | 75.86 | 67.33% to 82.74% | 32.97 | 24.17% to 43.14% | 1.132 |
| > 85.35 | 75.00 | 66.40% to 81.99% | 32.97 | 24.17% to 43.14% | 1.119 |
| > 88.07 | 75.00 | 66.40% to 81.99% | 34.07 | 25.15% to 44.27% | 1.138 |
| > 89.85 | 75.00 | 66.40% to 81.99% | 35.16 | 26.14% to 45.39% | 1.157 |
| > 92.94 | 74.14 | 65.49% to 81.24% | 35.16 | 26.14% to 45.39% | 1.143 |
| > 95.50 | 74.14 | 65.49% to 81.24% | 36.26 | 27.13% to 46.51% | 1.163 |
| > 96.84 | 74.14 | 65.49% to 81.24% | 37.36 | 28.12% to 47.62% | 1.184 |
| > 98.45 | 73.28 | 64.57% to 80.49% | 37.36 | 28.12% to 47.62% | 1.170 |
| > 102.7 | 72.41 | 63.66% to 79.73% | 37.36 | 28.12% to 47.62% | 1.156 |
| > 105.6 | 71.55 | 62.75% to 78.97% | 37.36 | 28.12% to 47.62% | 1.142 |
| > 105.9 | 71.55 | 62.75% to 78.97% | 38.46 | 29.13% to 48.73% | 1.163 |
| > 107.0 | 71.55 | 62.75% to 78.97% | 39.56 | 30.13% to 49.83% | 1.184 |
| > 107.9 | 71.55 | 62.75% to 78.97% | 40.66 | 31.14% to 50.93% | 1.206 |
| > 108.8 | 71.55 | 62.75% to 78.97% | 41.76 | 32.16% to 52.02% | 1.229 |
| > 111.2 | 70.69 | 61.85% to 78.20% | 41.76 | 32.16% to 52.02% | 1.214 |
| > 113.1 | 69.83 | 60.95% to 77.43% | 41.76 | 32.16% to 52.02% | 1.199 |
| > 114.1 | 69.83 | 60.95% to 77.43% | 42.86 | 33.18% to 53.11% | 1.222 |
| > 115.2 | 68.97 | 60.05% to 76.66% | 42.86 | 33.18% to 53.11% | 1.207 |
| > 115.7 | 68.10 | 59.16% to 75.89% | 42.86 | 33.18% to 53.11% | 1.192 |
| > 116.2 | 67.24 | 58.27% to 75.11% | 42.86 | 33.18% to 53.11% | 1.177 |
| > 116.9 | 67.24 | 58.27% to 75.11% | 43.96 | 34.21% to 54.19% | 1.200 |
| > 118.5 | 66.38 | 57.38% to 74.33% | 43.96 | 34.21% to 54.19% | 1.184 |
| > 119.8 | 66.38 | 57.38% to 74.33% | 45.05 | 35.24% to 55.27% | 1.208 |
| > 123.3 | 65.52 | 56.50% to 73.54% | 45.05 | 35.24% to 55.27% | 1.192 |
| > 127.3 | 65.52 | 56.50% to 73.54% | 46.15 | 36.28% to 56.34% | 1.217 |
| > 129.4 | 64.66 | 55.61% to 72.76% | 46.15 | 36.28% to 56.34% | 1.201 |
| > 131.7 | 63.79 | 54.74% to 71.97% | 46.15 | 36.28% to 56.34% | 1.185 |
| > 135.3 | 63.79 | 54.74% to 71.97% | 47.25 | 37.32% to 57.41% | 1.209 |
| > 140.2 | 63.79 | 54.74% to 71.97% | 48.35 | 38.36% to 58.48% | 1.235 |
| > 142.6 | 62.93 | 53.86% to 71.17% | 48.35 | 38.36% to 58.48% | 1.218 |
| > 143.9 | 62.93 | 53.86% to 71.17% | 49.45 | 39.41% to 59.54% | 1.245 |
| > 147.0 | 62.93 | 53.86% to 71.17% | 50.55 | 40.46% to 60.59% | 1.273 |
| > 149.5 | 62.93 | 53.86% to 71.17% | 51.65 | 41.52% to 61.64% | 1.302 |
| > 150.6 | 62.93 | 53.86% to 71.17% | 52.75 | 42.59% to 62.68% | 1.332 |
| > 152.7 | 62.07 | 52.99% to 70.38% | 52.75 | 42.59% to 62.68% | 1.314 |
| > 157.5 | 61.21 | 52.12% to 69.58% | 52.75 | 42.59% to 62.68% | 1.295 |
| > 161.1 | 60.34 | 51.25% to 68.78% | 52.75 | 42.59% to 62.68% | 1.277 |
| > 163.3 | 59.48 | 50.38% to 67.97% | 52.75 | 42.59% to 62.68% | 1.259 |
| > 165.8 | 58.62 | 49.52% to 67.17% | 52.75 | 42.59% to 62.68% | 1.241 |
| > 168.6 | 58.62 | 49.52% to 67.17% | 53.85 | 43.66% to 63.72% | 1.270 |
| > 171.2 | 57.76 | 48.66% to 66.36% | 53.85 | 43.66% to 63.72% | 1.251 |
| > 171.7 | 57.76 | 48.66% to 66.36% | 54.95 | 44.73% to 64.76% | 1.282 |
| > 171.8 | 56.90 | 47.81% to 65.54% | 54.95 | 44.73% to 64.76% | 1.263 |
| > 172.2 | 56.90 | 47.81% to 65.54% | 56.04 | 45.81% to 65.79% | 1.294 |
| > 172.8 | 56.03 | 46.95% to 64.73% | 56.04 | 45.81% to 65.79% | 1.275 |
| > 175.1 | 55.17 | 46.10% to 63.91% | 56.04 | 45.81% to 65.79% | 1.255 |
| > 178.2 | 54.31 | 45.25% to 63.09% | 56.04 | 45.81% to 65.79% | 1.236 |
| > 179.6 | 54.31 | 45.25% to 63.09% | 57.14 | 46.89% to 66.82% | 1.267 |
| > 182.9 | 54.31 | 45.25% to 63.09% | 58.24 | 47.98% to 67.84% | 1.301 |
| > 186.6 | 54.31 | 45.25% to 63.09% | 59.34 | 49.07% to 68.86% | 1.336 |
| > 187.6 | 53.45 | 44.41% to 62.27% | 59.34 | 49.07% to 68.86% | 1.315 |
| > 188.6 | 52.59 | 43.56% to 61.44% | 59.34 | 49.07% to 68.86% | 1.293 |
| > 191.0 | 52.59 | 43.56% to 61.44% | 60.44 | 50.17% to 69.87% | 1.329 |
| > 197.1 | 51.72 | 42.72% to 60.62% | 60.44 | 50.17% to 69.87% | 1.307 |
| > 205.6 | 51.72 | 42.72% to 60.62% | 61.54 | 51.27% to 70.87% | 1.345 |
| > 211.3 | 50.86 | 41.88% to 59.79% | 61.54 | 51.27% to 70.87% | 1.322 |
| > 214.6 | 50.86 | 41.88% to 59.79% | 62.64 | 52.38% to 71.88% | 1.361 |
| > 218.8 | 50.00 | 41.05% to 58.95% | 62.64 | 52.38% to 71.88% | 1.338 |
| > 222.3 | 50.00 | 41.05% to 58.95% | 63.74 | 53.49% to 72.87% | 1.379 |
| > 225.5 | 49.14 | 40.21% to 58.12% | 63.74 | 53.49% to 72.87% | 1.355 |
| > 229.1 | 48.28 | 39.38% to 57.28% | 63.74 | 53.49% to 72.87% | 1.331 |
| > 230.3 | 48.28 | 39.38% to 57.28% | 64.84 | 54.61% to 73.86% | 1.373 |
| > 230.4 | 47.41 | 38.56% to 56.44% | 64.84 | 54.61% to 73.86% | 1.348 |
| > 231.8 | 47.41 | 38.56% to 56.44% | 65.93 | 55.73% to 74.85% | 1.392 |
| > 235.3 | 46.55 | 37.73% to 55.59% | 65.93 | 55.73% to 74.85% | 1.367 |
| > 239.0 | 46.55 | 37.73% to 55.59% | 67.03 | 56.86% to 75.83% | 1.412 |
| > 240.7 | 46.55 | 37.73% to 55.59% | 68.13 | 57.99% to 76.80% | 1.461 |
| > 245.8 | 45.69 | 36.91% to 54.75% | 68.13 | 57.99% to 76.80% | 1.434 |
| > 252.8 | 45.69 | 36.91% to 54.75% | 69.23 | 59.13% to 77.77% | 1.485 |
| > 255.2 | 45.69 | 36.91% to 54.75% | 70.33 | 60.28% to 78.74% | 1.540 |
| > 256.0 | 44.83 | 36.09% to 53.90% | 70.33 | 60.28% to 78.74% | 1.511 |
| > 258.0 | 43.10 | 34.46% to 52.19% | 70.33 | 60.28% to 78.74% | 1.453 |
| > 261.8 | 43.10 | 34.46% to 52.19% | 71.43 | 61.43% to 79.69% | 1.509 |
| > 266.2 | 43.10 | 34.46% to 52.19% | 72.53 | 62.59% to 80.64% | 1.569 |
| > 270.6 | 42.24 | 33.64% to 51.34% | 72.53 | 62.59% to 80.64% | 1.538 |
| > 277.0 | 41.38 | 32.83% to 50.48% | 72.53 | 62.59% to 80.64% | 1.506 |
| > 282.6 | 41.38 | 32.83% to 50.48% | 73.63 | 63.75% to 81.59% | 1.569 |
| > 286.2 | 41.38 | 32.83% to 50.48% | 74.73 | 64.92% to 82.53% | 1.637 |
| * > 290.9 | 41.38 | 32.83% to 50.48% | 75.82 | 66.10% to 83.46% | 1.712 |
| > 294.1 | 40.52 | 32.03% to 49.62% | 75.82 | 66.10% to 83.46% | 1.676 |
| > 300.3 | 39.66 | 31.22% to 48.75% | 75.82 | 66.10% to 83.46% | 1.640 |
| > 306.5 | 38.79 | 30.42% to 47.88% | 75.82 | 66.10% to 83.46% | 1.605 |
| > 313.6 | 37.93 | 29.62% to 47.01% | 75.82 | 66.10% to 83.46% | 1.569 |
| > 319.8 | 37.07 | 28.83% to 46.14% | 75.82 | 66.10% to 83.46% | 1.533 |
| > 322.0 | 37.07 | 28.83% to 46.14% | 78.02 | 68.48% to 85.30% | 1.687 |
| > 325.3 | 36.21 | 28.03% to 45.26% | 78.02 | 68.48% to 85.30% | 1.647 |
| > 326.8 | 35.34 | 27.24% to 44.39% | 78.02 | 68.48% to 85.30% | 1.608 |
| > 329.3 | 34.48 | 26.46% to 43.50% | 78.02 | 68.48% to 85.30% | 1.569 |
| > 339.7 | 34.48 | 26.46% to 43.50% | 79.12 | 69.68% to 86.21% | 1.652 |
| > 348.0 | 33.62 | 25.67% to 42.62% | 79.12 | 69.68% to 86.21% | 1.610 |
| > 350.2 | 32.76 | 24.89% to 41.73% | 79.12 | 69.68% to 86.21% | 1.569 |
| > 354.1 | 32.76 | 24.89% to 41.73% | 80.22 | 70.89% to 87.11% | 1.656 |
| > 358.2 | 31.90 | 24.11% to 40.84% | 80.22 | 70.89% to 87.11% | 1.613 |
| > 361.5 | 31.03 | 23.34% to 39.95% | 80.22 | 70.89% to 87.11% | 1.569 |
| > 363.3 | 30.17 | 22.57% to 39.05% | 80.22 | 70.89% to 87.11% | 1.525 |
| > 364.1 | 29.31 | 21.80% to 38.15% | 80.22 | 70.89% to 87.11% | 1.482 |
| > 367.7 | 28.45 | 21.03% to 37.25% | 80.22 | 70.89% to 87.11% | 1.438 |
| > 377.7 | 28.45 | 21.03% to 37.25% | 81.32 | 72.10% to 88.00% | 1.523 |
| > 385.2 | 27.59 | 20.27% to 36.34% | 81.32 | 72.10% to 88.00% | 1.477 |
| > 386.7 | 27.59 | 20.27% to 36.34% | 82.42 | 73.33% to 88.88% | 1.569 |
| > 388.0 | 26.72 | 19.51% to 35.43% | 82.42 | 73.33% to 88.88% | 1.520 |
| > 389.7 | 26.72 | 19.51% to 35.43% | 83.52 | 74.57% to 89.75% | 1.621 |
| > 392.8 | 25.86 | 18.76% to 34.51% | 83.52 | 74.57% to 89.75% | 1.569 |
| > 395.2 | 25.86 | 18.76% to 34.51% | 84.62 | 75.82% to 90.61% | 1.681 |
| > 396.0 | 25.00 | 18.01% to 33.60% | 84.62 | 75.82% to 90.61% | 1.625 |
| > 397.8 | 24.14 | 17.26% to 32.67% | 84.62 | 75.82% to 90.61% | 1.569 |
| > 399.4 | 24.14 | 17.26% to 32.67% | 85.71 | 77.08% to 91.46% | 1.690 |
| > 400.0 | 23.28 | 16.52% to 31.75% | 85.71 | 77.08% to 91.46% | 1.629 |
| > 401.4 | 22.41 | 15.78% to 30.82% | 85.71 | 77.08% to 91.46% | 1.569 |
| > 408.1 | 22.41 | 15.78% to 30.82% | 86.81 | 78.35% to 92.29% | 1.700 |
| > 424.5 | 21.55 | 15.05% to 29.88% | 86.81 | 78.35% to 92.29% | 1.634 |
| > 436.4 | 20.69 | 14.32% to 28.94% | 86.81 | 78.35% to 92.29% | 1.569 |
| > 448.7 | 19.83 | 13.59% to 28.00% | 86.81 | 78.35% to 92.29% | 1.504 |
| > 462.5 | 19.83 | 13.59% to 28.00% | 87.91 | 79.64% to 93.11% | 1.640 |
| > 468.2 | 18.97 | 12.87% to 27.05% | 87.91 | 79.64% to 93.11% | 1.569 |
| > 471.7 | 18.10 | 12.16% to 26.10% | 87.91 | 79.64% to 93.11% | 1.498 |
| > 474.3 | 18.10 | 12.16% to 26.10% | 89.01 | 80.94% to 93.92% | 1.647 |
| > 483.0 | 17.24 | 11.45% to 25.14% | 89.01 | 80.94% to 93.92% | 1.569 |
| > 490.5 | 17.24 | 11.45% to 25.14% | 90.11 | 82.26% to 94.71% | 1.743 |
| > 494.9 | 16.38 | 10.74% to 24.17% | 90.11 | 82.26% to 94.71% | 1.656 |
| > 498.7 | 16.38 | 10.74% to 24.17% | 91.21 | 83.60% to 95.48% | 1.863 |
| > 505.1 | 16.38 | 10.74% to 24.17% | 92.31 | 84.96% to 96.22% | 2.129 |
| > 514.1 | 16.38 | 10.74% to 24.17% | 93.41 | 86.35% to 96.94% | 2.484 |
| > 517.3 | 15.52 | 10.05% to 23.20% | 93.41 | 86.35% to 96.94% | 2.353 |
| > 519.2 | 14.66 | 9.356% to 22.22% | 93.41 | 86.35% to 96.94% | 2.223 |
| > 523.3 | 13.79 | 8.672% to 21.24% | 93.41 | 86.35% to 96.94% | 2.092 |
| > 541.9 | 12.93 | 7.995% to 20.24% | 93.41 | 86.35% to 96.94% | 1.961 |
| > 562.3 | 12.07 | 7.327% to 19.24% | 93.41 | 86.35% to 96.94% | 1.830 |
| > 574.9 | 12.07 | 7.327% to 19.24% | 94.51 | 87.78% to 97.63% | 2.197 |
| > 584.3 | 11.21 | 6.667% to 18.23% | 94.51 | 87.78% to 97.63% | 2.040 |
| > 585.4 | 10.34 | 6.017% to 17.21% | 94.51 | 87.78% to 97.63% | 1.883 |
| > 606.2 | 9.483 | 5.378% to 16.19% | 94.51 | 87.78% to 97.63% | 1.726 |
| > 631.3 | 8.621 | 4.750% to 15.14% | 94.51 | 87.78% to 97.63% | 1.569 |
| > 652.2 | 8.621 | 4.750% to 15.14% | 95.60 | 89.24% to 98.28% | 1.961 |
| > 676.6 | 8.621 | 4.750% to 15.14% | 96.70 | 90.75% to 99.10% | 2.615 |
| > 697.0 | 8.621 | 4.750% to 15.14% | 97.80 | 92.34% to 99.61% | 3.922 |
| > 729.8 | 7.759 | 4.135% to 14.09% | 97.80 | 92.34% to 99.61% | 3.530 |
| > 765.3 | 6.897 | 3.536% to 13.02% | 97.80 | 92.34% to 99.61% | 3.138 |
| > 820.4 | 6.034 | 2.954% to 11.93% | 97.80 | 92.34% to 99.61% | 2.746 |
| > 873.8 | 5.172 | 2.392% to 10.83% | 97.80 | 92.34% to 99.61% | 2.353 |
| > 915.3 | 4.310 | 1.855% to 9.695% | 97.80 | 92.34% to 99.61% | 1.961 |
| > 971.7 | 3.448 | 1.349% to 8.532% | 97.80 | 92.34% to 99.61% | 1.569 |
| > 1019 | 2.586 | 0.7049% to 7.329% | 97.80 | 92.34% to 99.61% | 1.177 |
| > 1096 | 2.586 | 0.7049% to 7.329% | 98.90 | 94.04% to 99.94% | 2.353 |
| > 1202 | 1.724 | 0.3063% to 6.069% | 98.90 | 94.04% to 99.94% | 1.569 |
| > 1272 | 0.8621 | 0.04422% to 4.722% | 98.90 | 94.04% to 99.94% | 0.7845 |
| > 1329 | 0.8621 | 0.04422% to 4.722% | 100.0 | 95.95% to 100.0% |  |

**Youden’s index cut-off*

*Sensitivity (Sn), specificity (Sp), positive and negative predictive values (PPV and NPV,*

*respectively), and positive likelihood ratis (LR+)*


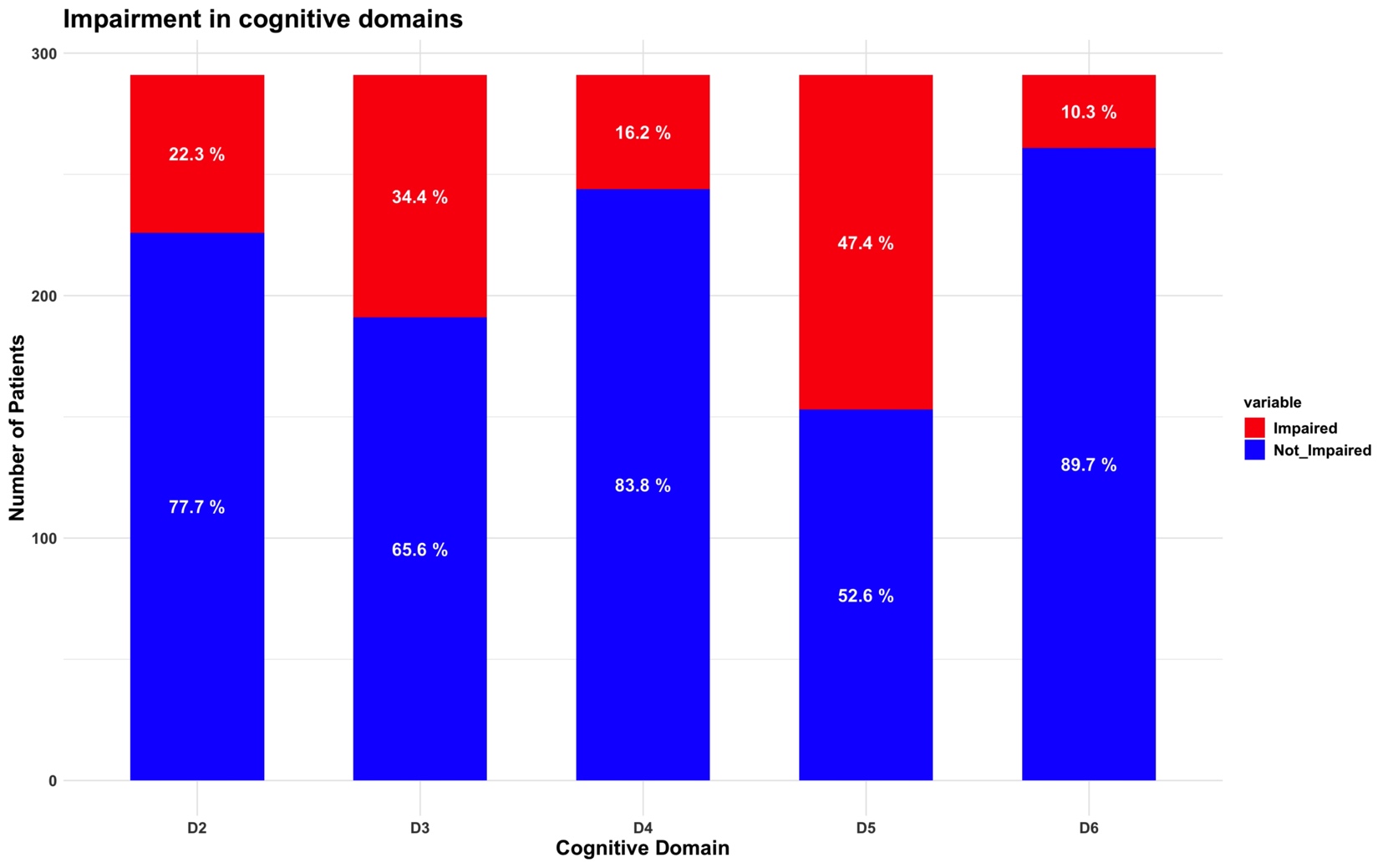


**Supplementary Figure 1. Proportion of patients with impairment in each ACR-NB domain.**

**Supplementary Figure 2. Serum levels of IL-6 and TNF-α, in patients with and without impairment for each of the ACR-NB domains.** Strip plots with median bars showing, from left to right, results for patients with no impairment and impairment in the domain. Each circle represents a single subject, with the top of the bar indicating the median for the subjects and error bars denoting the interquartile ranges. Statistical significance was determined using a Bonferroni-corrected Mann-Whitney *U* test with significant differences indicated by asterisks (** p ≤ 0.01).

**Supplementary Figure 3. Serum levels of various analytes in SLE patients with and without impairment for each of the ACR-NB domains.** Strip plots with median bars showing, from left to right, results for patients with no impairment and impairment in the domain. Each circle represents a single subject, with the top of the bar indicating the median for the subjects and error bars denoting the interquartile ranges. Statistical significance was determined using a Bonferroni-corrected Mann-Whitney *U* test.

**Supplementary Figure 4**. Graphs illustrating the linear relationship between serum S100A8/A9 levels and the z score for individual tests within the domains of the ACR-NB that showed a statistically significant relationship through regression analysis.

**Supplementary Figure 5. Matrix displaying significant correlations among the different analytes, inflammatory markers, and disease activity.** Correlations were determined by the Spearman’s rank correlation coefficient. The size of the dots indicates the p-value and the color intensity the strength of the correlation. Non-significant correlations (p ≥ 0.05) are not displayed. Disease activity as determined by the SLEDAI-2K. Inflammatory markers median (IQR): Hs-CRP 1.65 mg/L (0.66 – 3.28) and ESR 18 (9-29) mm/hr.
